# Identification of GSN and LAMC2 as Key Prognostic Genes of Bladder Cancer by Integrated Bioinformatics Analysis

**DOI:** 10.3390/cancers12071809

**Published:** 2020-07-06

**Authors:** Jia-Lian Yang, Charles C. N. Wang, Jia-Hua Cai, Che-Yi Chou, Yu-Chao Lin, Chin-Chuan Hung

**Affiliations:** 1 Department of Pharmacy, College of Pharmacy, China Medical University, Taichung 404, Taiwan; u9903101@gmail.com; 2 Department of Bioinformatics and Medical Engineering, Asia University, Taichung 41354, Taiwan; cnwang@asia.edu.tw (C.C.N.W.); as6309123@yahoo.com.tw (J.-H.C.); 3 Division of Nephrology, Asia University Hospital, Taichung 41354, Taiwan; cychou.chou@gmail.com; 4 Department of Post-Baccalaureate Veterinary Medicine, Asia University, Taichung 413, Taiwan; 5 Graduate Institute of Clinical Medical Science, China Medical University, Taichung 404, Taiwan; cmchlyc@yahoo.com.tw; 6 Division of Pulmonary and Critical Care Medicine, Department of Internal Medicine, China Medical University Hospital, Taichung 404, Taiwan; 7 Department of Pharmacy, China Medical University Hospital, Taichung 404, Taiwan

**Keywords:** bladder cancer, weighted gene coexpression network analysis, prognosis, GEO, TCGA, HPA

## Abstract

Bladder cancer is a common malignancy with mechanisms of pathogenesis and progression. This study aimed to identify the prognostic hub genes, which are the central modulators to regulate the progression and proliferation in the specific subtype of bladder cancer. The identification of the candidate hub gene was performed by weighted gene co-expression network analysis to construct a free-scale gene co-expression network. The gene expression profile of GSE97768 from the Gene Expression Omnibus database was used. The association between prognosis and hub gene was evaluated by The Cancer Genome Atlas database. Four gene-expression modules were significantly related to bladder cancer disease: the red module (human adenocarcinoma lymph node metastasis), the darkturquioise module (grade 2 carcinoma), the lightgreen module (grade 3 carcinoma), and the royalblue module (transitional cell carcinoma lymphatic metastasis). Based on betweenness centrality and survival analysis, we identified laminin subunit gamma-2 (LAMC2) in the grade 2 carcinoma, gelsolin (GSN) in the grade 3 carcinoma, and homeodomain-interacting protein kinase 2 (HIPK2) in the transitional cell carcinoma lymphatic metastasis. Subsequently, the protein levels of LAMC2 and GSN were respectively down-regulated and up-regulated in tumor tissue with the Human Protein Atlas (HPA) database. Our results suggested that LAMC2 and GSN are the central modulators to transfer information in the specific subtype of the disease.

## 1. Introduction

Bladder cancer ranks 13th in cancer death worldwide and has heterogeneous subtypes, in which the urothelial carcinoma is the most common [1,2]. According to the degree of muscle invasion and metastasis, urothelial carcinomas can be classified into three types: non-muscle invasive, muscle-invasive, and metastasis bladder cancer [2]. Resection and radical cystectomy are the mainstream treatment for non-muscle invasive and muscle-invasive bladder cancer, respectively. On the other hand, the cisplatin-based or gemcitabine-based combination therapies would be used as first-line treatment for metastasis or recurrent bladder cancer [3,4,5]. Most bladder patients were diagnosed with non-muscle invasive bladder cancer (NMIBC) at presentation and with favorable prognosis [2]. However, 50% of recurrences and 20% of case progressions were observed in 5 years for patients with NMIBC [6]. In addition, patients can still present a poor prognosis when treated with standard treatment or immune checkpoint inhibitors following the failure of the standard cancer treatment [3,4,5]. Although the fibroblast growth factor receptor (FGFR) inhibitor was newly approved in 2019 and proved to increase the survival rates, only patients with FGFR genetic alterations can be treated [7]. Other than patients with FGFR alteration, the molecular mechanisms of different subtypes of bladder cancer are still unknown. As a result, the identification of the key genes or biological processes that modulate the tumor proliferation and progression is important to realize molecular mechanisms of heterogeneous subtypes. Furthermore, the identified genes and mechanisms can be developed into biomarkers or treatments to improve the survival of patients with bladder cancer. Via connecting the clinical information with gene expression, the identified key genes will be helpful for survival prediction, as well as offering the potential mechanisms of progression [8,9,10,11,12]. Several bioinformatics methods have been proposed currently, such as protein–protein interaction, signaling networks, metabolic networks, gene regulatory networks, and co-expression networks. Among these bioinformatics analyses, the co-expression network can build type-specific networks with nearly comprehensive coverage of human genes [13]. In addition, it can estimate the degree of interconnectivity between genes and speculate the functionally related gene expression pattern [14]. Weighted gene co-expression network analysis (WGCNA), which is a powerful method to construct a co-expression network, has been widely used to process the analysis of large-scale datasets to recognize the highly correlated genes [15]. Subsequently, according to clinical information, WGCNA can identify the candidate biomarker whose gene expression level is highly associated with the survival or progression of cancer [16,17,18,19]. In this study, WGCNA would be performed to identify the key modulators in the different morphologic types of bladder cancer based on the transcriptional profile of a bladder cancer cell line from GSE97768. The Cancer Genome Atlas (TCGA), which is a project supervised by the National Cancer Institute (NCI) and National Human Genome Research Institute (NHGRI) would be used to find the potential prognostic biomarker of bladder cancer [20].

## 2. Results

### 2.1. Weighted Co-Expression Network Construction and Key Modules Identification

**Figure 1:**
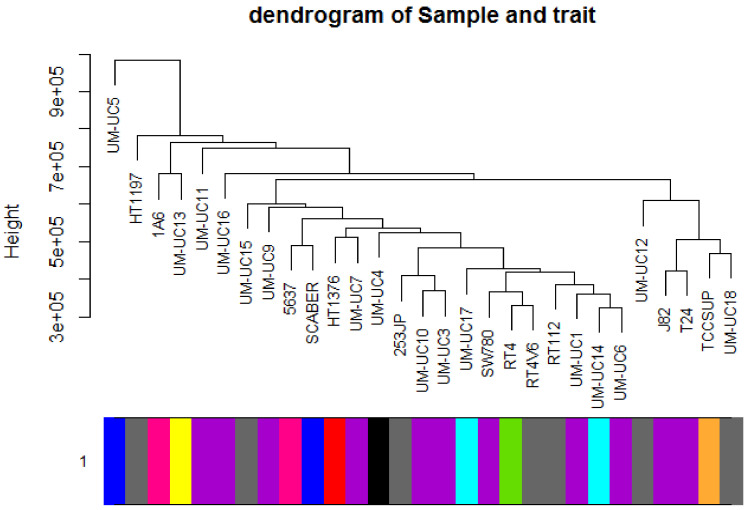
Clustering dendrogram of 29 samples.

Twenty-nine cell lines with subtype data were included. A total of 1,231,947 genes were included to conduct the WGCNA analysis. The included samples were clustered with the average linkage hierarchical clustering method (Figure 1). 

**Figure 2:**
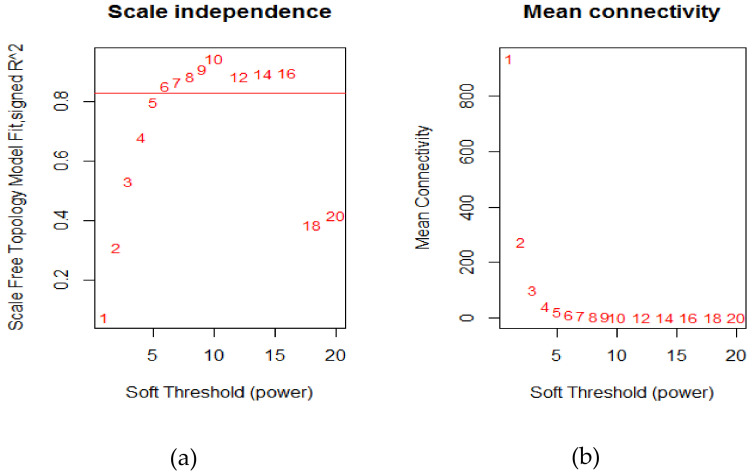
Determination of soft-thresholding power in weighted gene co-expression network analysis (WGCNA) analysis; (**a**) The scale-free fit index for various soft-thresholding powers β; (**b**) The mean connectivity for various soft-thresholding powers.

The power of β = 6 (scale-free R2 = 0.9) was selected as the soft-thresholding parameter to conduct a scale-free network (Figure 2). 

**Figure 3:**
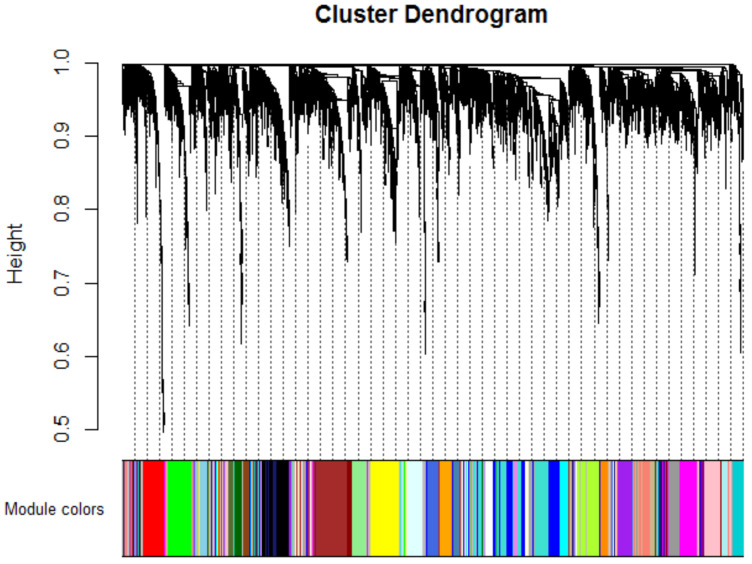
Dendrogram of all differentially expressed genes clustered based on a dissimilarity measure.

A total of 35 modules were identified with the average linkage hierarchical clustering (Figure 3).

The size of each identified module is listed in the Supplementary Materials (Table S1). 

**Figure 4:**
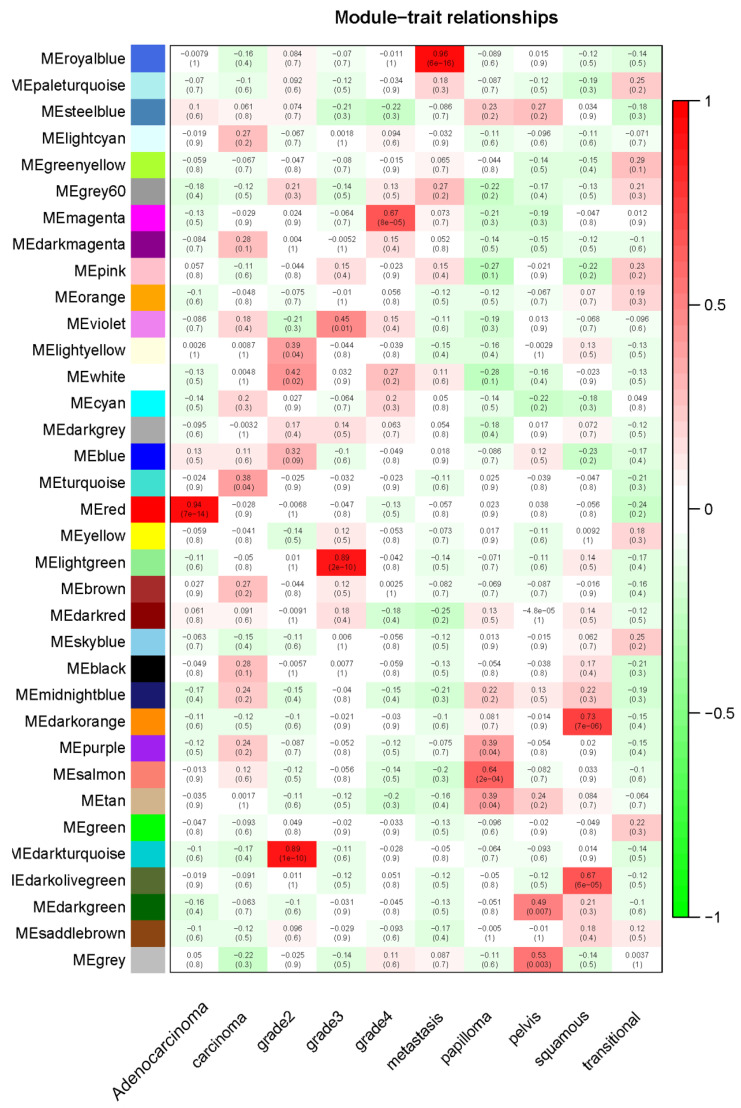
Heatmap of the correlation between module eigengenes and clinical subtypes of bladder cancer. Each column contained the corresponding correlation and *p* value.

Furthermore, the module eigengenes (MEs) of the red module, darkturquoise module, lightgreen module, magenta module, royalblue module, salmon module, gray module, and darkorange module were found to have the highest correlation with subtypes (human adenocarcinoma lymph node metastasis, grade 2 carcinoma, grade 3 carcinoma, grade 4 carcinoma, transitional cell carcinoma lymphatic metastasis, transitional cell papilloma, transitional cell carcinoma of the renal pelvis, and squamous cell carcinoma) (Figure 4).

**Figure 5:**
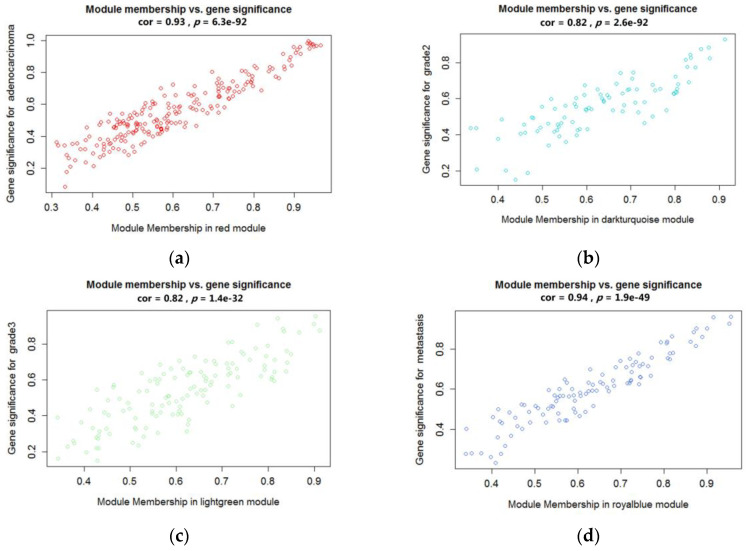
Scatter plots of the highly correlated module in different clinical subtypes of bladder cancer. (**a**) Red module was found to have the highest association with human adenocarcinoma lymph node metastasis; (**b**) Darkturquioise module was found to have the highest association with Grade 2 carcinoma; (**c**) Lightgreen module was found to have the highest association with Grade 3 carcinoma; (**d**) Royalblue module was found to have the highest association with transitional cell carcinoma lymphatic metastasis.

Those modules were selected as the clinical significant module for further analysis. Defined by module connectivity and measured by the absolute value of the Pearson’s correlation and clinical trait relationship, the red module, the lightgreen module, the magenta module, and the royalblue module were found to have the highest correlation with the subtypes (human adenocarcinoma lymph node metastasis, grade 2 carcinoma, grade 3 carcinoma, transitional cell carcinoma lymphatic metastasis) (Figure 5a–d). These modules were used to identify hug genes.

### 2.2. Functional and Pathway Enrichment Analysis

Hub genes were uploaded to FunRich (Table S2). Findings with higher scores are more significant than low-scoring results. Only significant hits with overlap size ≥ 2 (genes that are overlapping in the same pathway) were considered. Gene Ontology (GO) analysis showed that genes of cell lines of human adenocarcinoma lymph node metastasis were enriched in the top 10 biological processes (BP) and molecular process (MP) (Figure S1A–C). Hub genes were related to the metabolism of lipids and the immune signaling pathway. Among the functional and pathway enrichment analysis, metabolism of lipids and lipoproteins, T-cell receptor (TCR) signaling in CD4+ T cells, and CXCR4 (CXC receptor 4)-mediated signaling events were enriched most. The abnormal metabolism of lipids and lipoproteins has been presented in the environment of the tumor. To maintain the viability, the tumor scavenged extracellular desaturated fatty acids, which were responsible for rescuing the unfolded protein response and cell death [21,22,23]. On the other hand, in the anti-cancer mechanism, TCR signaling would initiate the intracellular signals to activate the anti-cancer responses of T cells. The alteration or abnormalities of TCR signaling leads to the defect of the immune response to tumor [24]. In addition to TCR signaling pathways, the binding of CXCR4 and chemokine CXCL12 activated several pathways involved in the molecular mechanism of progression, angiogenesis, and metastasis in tumor [25]. In the cell lines of grade 2 carcinoma, the top 10 biological processes (BP) and molecular process (MP) were shown in Figure S2. Hub genes were involved in complex processes and can be associated with the integrin-related pathway and cell mitosis process 16. Integrin-related pathways performed a function in the progression, angiogenesis, and metastasis of solid tumors. Alterations of cell mitosis, which is one of the cell cycle processes, would lead to abnormalities of carcinogenesis [26,27]. On the other hand, hub genes identified in the grade 3 carcinoma were also involved in the several molecular processes, which were associated with platelet-derived growth factor (PDGF), insulin-like growth factor 1 (IGF-1) pathways, and tumor necrosis factor (TNF)-related apoptosis-inducing ligand (TRAIL) pathways (Figure S3). Both PDGF and IGF-1 signaling pathways contributed to tumor growth, angiogenesis, and metastatic activities in various cancers as binding to the receptors [28]. Besides, TRAIL pathways were a potentially targeted pathway, which induced apoptosis and necrosis of tumor cells by intracellular signaling pathways including NFκB, MAPK/ERK, and PI3K/AKT. Inactivation of the TRAIL-related pathways might have an impact on the tumor onset and progression [29]. In the cell lines of transitional cell carcinoma lymphatic metastasis, enrichment of DNA damage response (DDR) and endothelial NOS (eNOS) activity were found in the top 10 biological processes (BP) and molecular process (MP). The results of the function and pathway analysis were also confirmed (Figure S4 A–C). DDR contained the ataxia-telangiectasia mutated (ATM) and ATM and RAD3-related (ATR) kinases, which promoted the cell cycle arrest and induced DNA repair. Increasing DDR signaling was associated with the resistance to genotoxic therapies [30]. Besides, eNOS activity was suggested, which could promote the metastasis with activating the angiogenesis, blocking apoptosis, or driving invasion via several molecular pathways [31].

### 2.3. Real Hub Genes Identification and Validation

**Figure 6:**
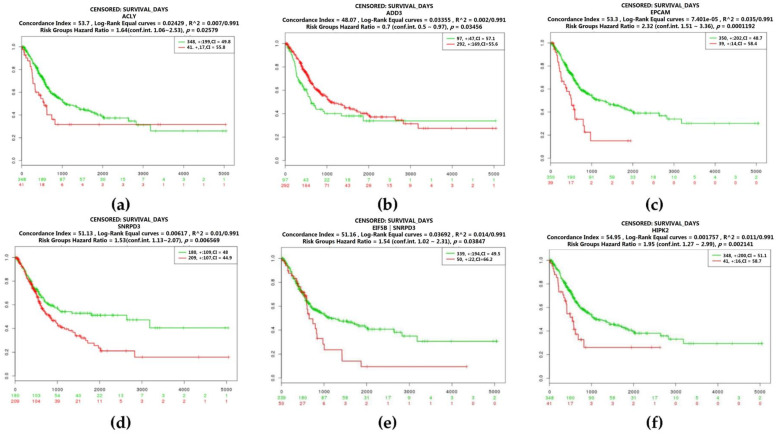
Overall survival of the hub genes in bladder cancer with human adenocarcinoma lymph node metastasis and transitional cell carcinoma lymphatic metastasis based on Kaplan–Meier plotter. The horizontal axis represents the time to event. The patients were stratified into the high-level group and low-level group and labeled with color. The red line indicates the samples with high risk, and the green line shows the samples with low risk. *p* < 0.05 is considered a statistically significant difference in mortality between the high-level group and low-level group. (**a**)–(**e**) Hub genes identified in human adenocarcinoma lymph node metastasis; (**f**) Hub genes identified in transitional cell carcinoma lymphatic metastasis. (**a**) ATP citrate lyase (ACLY); (**b**) Adducin 3 (ADD3); (**c**) epithelial cell adhesion molecule (EPCAM); (**d**) small nuclear ribonucleoprotein D3 polypeptide (SNRPD3); (**e**) Co-expression of EIF5B and SNRD3; (**f**) Homeodomain-interacting protein kinase 2 (HIPK2).

We also analyze the prognosis of gene expression in bladder cancer. Kaplan–Meier survival curves by different mRNA expression levels of hub genes were plotted. The *p*-values of the log-rank test and concordance index (CI) were estimated. CI was the estimated probability that patients with a higher risk will experience the event after those with a lower risk. The *p*-value less than 0.05 indicated a statistically significant difference in mortality between the high-level group and low-level group. Four genes were related to the survival of bladder cancer with human adenocarcinoma lymph node metastasis. The high expression level of ATP citrate lyase (ACLY) (log-rank test: *p* = 0.02579) and adducin 3 (ADD3) (log-rank test: *p* = 0.03456) demonstrated poor prognosis, and patients with the low expression level of epithelial cell adhesion molecule (EPCAM) (log-rank test: *p* = 0.0001192) and small nuclear ribonucleoprotein D3 polypeptide (SNRPD3) (log-rank test: *p* = 0.006569) had a higher risk of mortality. Besides, patients with the reduced expression level of the hug gene, EIF5B, and an increasing expression level of SNRPD3 had a poor prognosis (log-rank test: *p* = 0.03847) (Figure 6). On the other hand, homeodomain interacting protein kinase 2 (HIPK2) was identified in bladder cancer with transitional cell carcinoma lymphatic metastasis. The higher expression level of HIPK2 was related to a higher risk of mortality (log-rank test: *p* = 0.002141) (Figure 6). 

**Figure 7:**
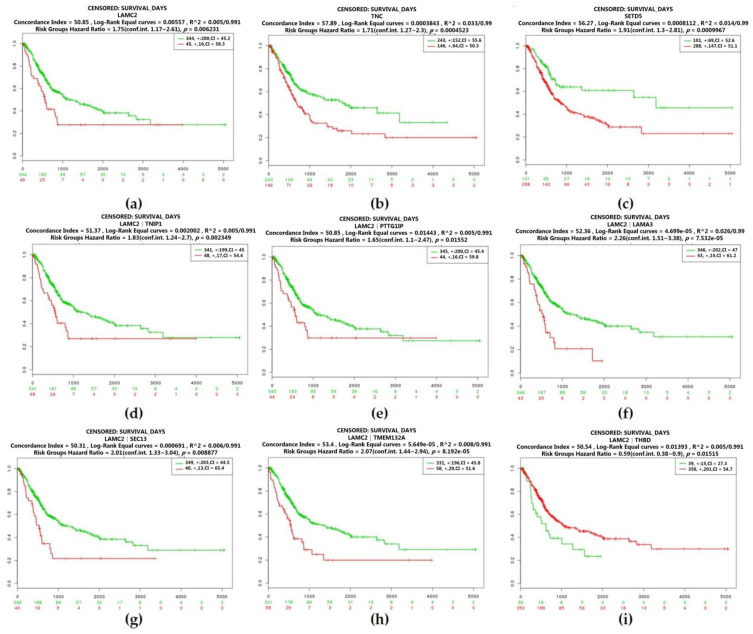
Overall survival of the co-expression of hub genes of bladder cancer with grade 2 carcinoma based on Kaplan–Meier plotter. The horizontal axis represents the time to event. The patients were stratified into the high-level group and low-level group and labeled with color. The red line indicates the samples with high risk, and the green line shows the samples with low risk. *p* < 0.05 is considered a statistically significant difference in mortality between the high-level group and low-level group. (**a**) Laminin subunit gamma 2 (LAMC2); (**b**) Tenascin C (TNC); (c) SET domain containing 5 (SETD5); (**d**) Co-expression of LAMC2 and TNIP1; (**e**) Co-expression of LAMC2 and PTTG1IP; (**f**) Co-expression of LAMC2 and LAMA3; (**g**) Co-expression of LAMC2 and SEC3; (**h**) Co-expression of LAMC2 and TMEM132A; (**i**) Co-expression of LAMC2 and THBD. Abbreviation: TNIP1 *TNFAIP3 Interacting Protein 1*, PTTG1IP *Prostaglandin-endoperoxide synthase 1*, LAMA3 *Laminin subunit alpha-3*, SEC3 *Exocyst complex component 1*, TMEM132A *Transmembrane protein 132A*, THBD *Thrombomodulin*.

Besides, we plotted the Kaplan–Meier survival curves by different mRNA expression levels of hub genes of the bladder cancer with grade 2 carcinoma. Three genes were associated with survival, including laminin subunit gamma 2 (LAMC2), tenascin C (TNC), and SET domain containing 5 (SETD5). Reduction in the expression level of LAMC2 (log-rank test: *p* = 0.006231) or SETD5 (log-rank test: *p* = 0.0009967) was accompanied by poor survival benefit (0.002349), while patients with reducing the expression level of TNC (log-rank test: *p* = 0.0004523) had a better prognosis. Moreover, hub genes (PTTG1IP, LAMA3, TNIP1, TMEM132A, SEC13, and THBD) identified as co-expressed with LAMC2 also increase the risk of mortality (log-rank test: *p* = 0.01552; log-rank test: *p* = 7.532 × 10^−5^; log-rank test: *p* = 0.002349; log-rank test: *p* = 8.192 × 10^−5^; log-rank test: *p* = 0.0008877; log-rank test: *p* = 0.01515) (Figure 7).

**Figure 8:**
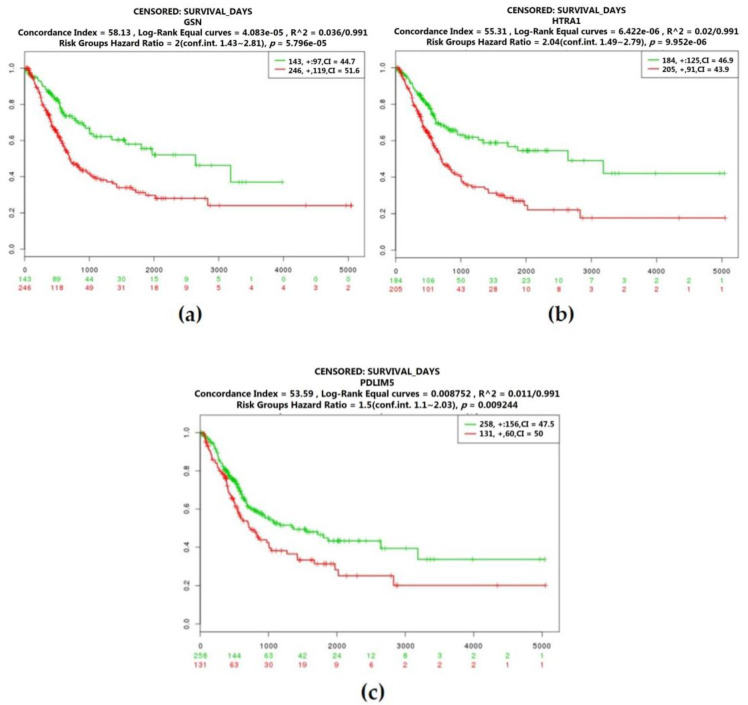
Overall survival of the co-expression of hub genes of bladder cancer with grade 3 carcinoma based on Kaplan–Meier plotter. The horizontal axis represents the time to event. The patients were stratified into the high-level group and low-level group and labeled with color. The red line indicates the samples with high risk, and the green line shows the samples with low risk. *p* < 0.05 is considered a statistically significant difference in mortality between the high-level group and low-level group. (**a**) Gelsolin (GSN); (**b**) HtrA serine peptidase 1 (HTRA1); (**c**) PDZ and LIM domain 5 (PDLIM5).

According to the Kaplan–Meier survival curves by different mRNA expression levels of hub genes of the bladder cancer with grade 3 carcinoma, GSN, PDLIM4, and HTRA1 were identified as prognostic genes (log-rank test: *p* = 5.796 × 10^−5^; log-rank test: *p* = 9.952 × 10^−6^; log-rank test: *p* = 0.009244). With a high expression level of these genes, patients had a poor prognosis (Figure 8).

### 2.4. Identification of Candidate Genes with High Weighted Degree Score

**Figure 9:**
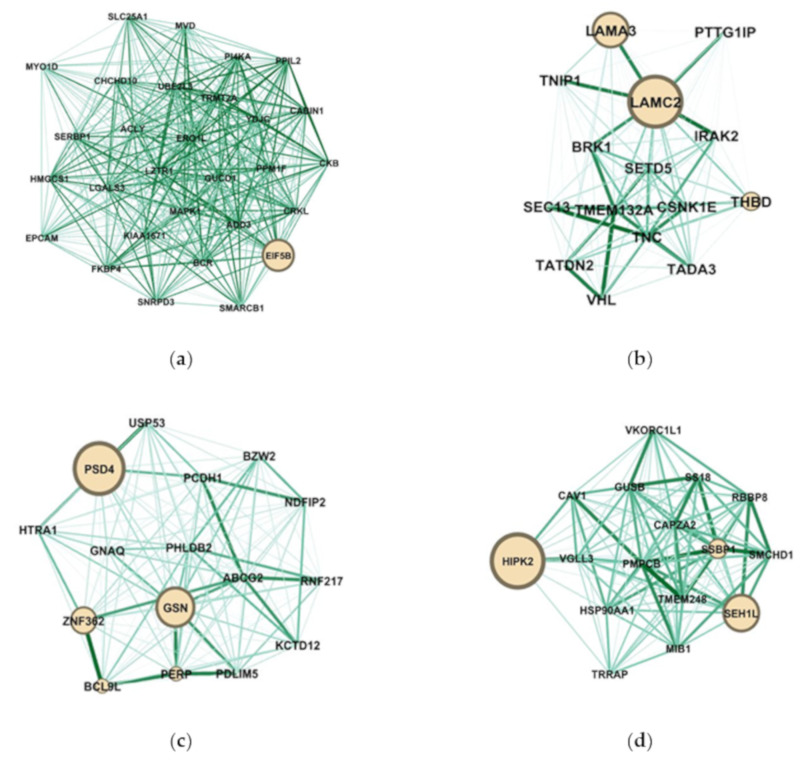
The network of hub genes in the red module, darkturquioise module, lightgreen module, and royalblue module. Nodes represent genes and node size indicates the betweenness score. Edges are colored by weight. (**a**) The network hub genes of human adenocarcinoma lymph node metastasis; (**b**) The network hub genes of grade 2 carcinoma; (**c**) The network hub genes of grade 3; (**d**) The network hub genes of transitional cell carcinoma lymphatic metastasis.

Highly connected hub genes were defined by module connectivity (ModuleMembership > 0.8) and clinical trait relationship (GeneSignificance > 0.2). The hub genes of each module were visualized as networks in Gephi and screened out the top candidate gene by in-rank ordering of betweenness score for further analysis (http://www.funrich.org/) (Figure 9).

Network analysis of the modules obtained from WGCNA was focused on the betweenness centrality (BC) of the genes within the modules. Since this measure reflects influence over the information transfer between different genes, we identified genes for which betweenness is considerably changed between the four networks (red module, darkturquioise module, lightgreen module, and royalblue module), as shown in Figure 9. Using the betweenness value to rank genes in the human adenocarcinoma lymph node metastasis network, we identified EIF5B as the gene with the highest betweenness (BC = 1.0), suggesting that it has a central role in information transfer in this module. The role of EIF5B in bladder cancer was unknown, but it was reported as an antagonist of the G0 phase. The overexpression of EIF5B caused cell death [32]. On the other hand, LAMA3 and LAMC2 were identified in the network analysis of the grade 2 carcinoma. The methylation of LAMA3 and LAMC2 were associated with poor prognosis in patients with muscle invasive bladder cancer (MIBC). Besides, the frequency of LAMC2 methylation was related to MIBC recurrence [33,34,35]. In the grade 3 carcinoma, PSD4 and GSN had the higher betweenness (BC = 1.0; BC = 0.193548). GSN mediating the level of the actin remodeling can induce the ATGF3 inhibiting the metastasis of bladder cancer cells [36]. According to the results of survival analysis, GSN has been suggested as the most potent regulator in bladder cancer with grade 3 carcinoma. The function of PSD4 was related to driving the cell line into epithelial-to-mesenchymal transition. In human breast tumors, the reduced expression of PSD4 was associated with increasing the poor prognosis and cancer stemness [37,38]. In the transitional cell carcinoma lymphatic metastasis, we identified HIPK2 and SEH1 like nucleoporin (SEH1L). It was reported that HIPK2 was the tumor suppressor, while SEH1L was correlated with the time of disease-free status in melanoma [39,40]. Based on the results of survival analysis, HIPK2 could be an important gene in bladder cancer with transitional cell carcinoma lymphatic metastasis.

### 2.5. The Protein Expression of Hub Genes

**Figure 10:**
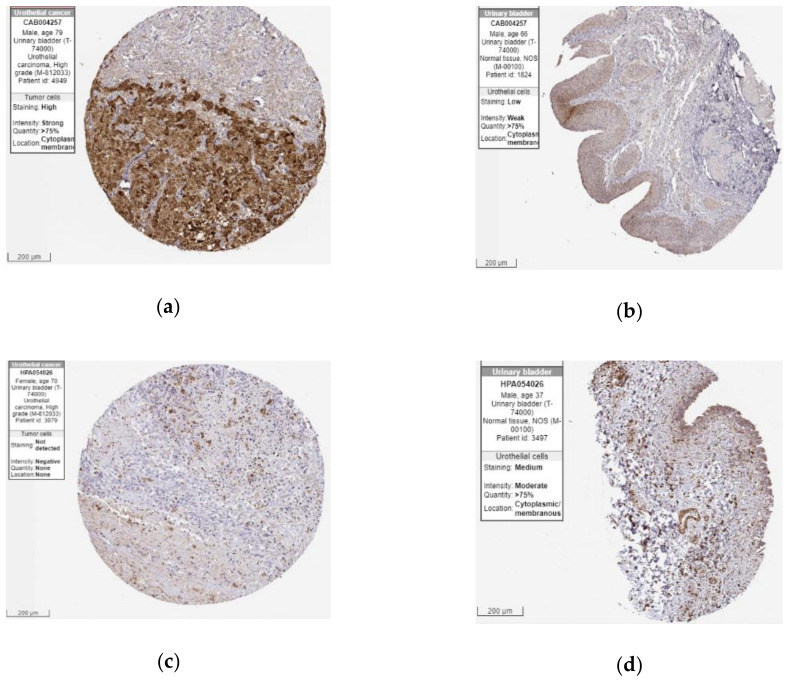
Immunohistochemistry of LAMC2 and GSN gene in bladder cancer (BLCA) and normal tissues from the Human Protein Atlas (HPA) database. (**a**) Protein levels of LAMC2 in BLCA tissues (antibody CAB004257; staining: high; intensity: strong; quantity: >75%); (**b**) Protein levels of LAMC2 in normal urinary bladder tissues (antibody CAB004257; staining: low; intensity: weak; quantity: >75%); (**c**) Protein levels of GSN in BLCA tissues (antibody HPA054026; staining: not detected; intensity: negative; quantity: none); (**d**) Protein levels of GSN in normal urinary bladder tissues (antibody HPA054026; staining: medium; intensity: moderate; quantity: >75%).

After the three candidate genes (GSN, LAMC2, HIPK1) were identified by verifying the expression levels in the TCGA database, we used the Human Protein Atlas (HPA) database to confirm the protein expression in urothelial normal tissue and urothelial tumor tissue. As shown in Figure 10, the protein expression of LAMC2 was lower in the tumor tissues compared to the normal tissue. On the other hand, the protein expression of GSN was moderately higher in tumor tissues than normal. Taken together, bladder cancer patients with a down-regulated level of LAMC2 or up-regulated level of GSN were related to poor prognosis (Figure 7, Figure 8 and Figure 10).

Conclusively, GSN and LAMC2 can be candidate genes for further investigation of the molecular mechanism on bladder cancer. The role of these prognostic genes in cancer was validated in other experimental articles, and the impact on tumorigenic is unclear. From the experiment results, GSN overexpressed would inhibit the wound-healing assay, which can assess the ability of migration of T24-L [36]. Besides, higher microsatellite alteration at GSN was only observed in bladder cancer patients [41]. Smith et al. observed a higher expression level of LAMC2 was observed in the liver and lung metastasis site of nude mice injected with T24T [33]. Besides, LAMC2 with a higher methylation level and lower expression level were presented in bladder patients [35]. Considering the high heterogeneity of bladder cancer, the small number of patients and the limited types of cancer cell lines lead to the conflict trend of expression of these genes in tumorigenesis and progression. Nevertheless, based on previous experimental results, the gene expression level of GSN and LAMC2 were novel prognostic biomarkers, which are also identified by a large clinical database (TGCA) and WGCNA analysis in our study. In other words, two identified can be applied from bioinformatic information to experimental results.

## 3. Discussion

Bladder cancer is a disease with high recurrence and variable prognosis. Although several prognostic models were proposed, most lacked accuracy [42,43]. Better and more accurate biomarkers for cancer-specific prognosis are highly needed. On the other hand, due to the high recurrence rate of the standard treatment of bladder, exploring the molecular mechanisms involved in the development and progression of bladder cancer is important. Here, we used an integrated analysis to screen the prognostic biomarkers and potential molecular pathways in different disease types.

With the WGCNA analysis, we identified potential genes related to the survival of bladder cancer. Among the modules, clinical characteristic correlated subtypes as follows: human adenocarcinoma lymph node metastasis, grade 2 carcinoma, grade 3 carcinoma, and transitional cell carcinoma lymphatic metastasis. Confirmed by the TCGA database, 11 hub genes were found. In human adenocarcinoma lymph node metastasis, EPCAM, ACLY, ADD3, and SNRPD3 were identified. On the other hand, three genes (LAMC2, TNC, and SETD5) were found related to the survival of bladder cancer in grade 2 carcinoma. GSN, HTRA1, and PDLIM5 were the prognostic genes in the grade 3 carcinoma. Moreover, we also found that patients with a high expression level of HIPK2 had a higher mortality risk. 

ATP citrate lyase (ACLY), epithelial cell adhesion molecule (EPCAM), HtrA Serine Peptidase 1 (HTRA1), and laminin subunit gamma 2 (LAMC2) were reported as a potential biomarker. ACLY is an enzyme to convert citrate to acetyl- coenzyme A, which is an essential precursor for lipid synthesis. The uptake or storage of lipid is important for cancer cell growth or migration. In addition, ACLY was found with a higher expression level in the tumor cells of bladder cancer than in the normal cells [44,45]. EPCAM was a transmembrane glycoprotein identified with higher expression level in the urine of patients with a higher grade or advanced stage of bladder cancer [46,47]. Serine protease HtrA1 was suggested to be related to TGF-βeta signaling pathways [48]. Down-regulation of HTRA1 in bladder cancer tissue regardless of grade and stage implicated that HTRA1 may act as a potential diagnostic biomarker of early stage [49]. TNC is an extracellular matrix protein involved in cell adhesion, migration, and growth. It is reported that the expression level of TNC was increasing in invasive tumor cells, but the prognostic significance of TNC in urothelial carcinoma (UC) needs further investigation [50,51]. Moreover, several cancer-related genes identified by us have been reported for other cancers, but not for bladder cancer. Small nuclear ribonucleoprotein D3 polypeptide (SNRPD3) encode the components of the spliceosome related to mRNA splicing. It has been suggested that SNRPD3 was associated with the aggressiveness of tumor cells [52]. PTTG1-interacting protein (PTTG1IP), which is an oncogenic protein regulated the cell cycle with PTTG1. In thyroid cancer, PTTG1IP acted as a suppressor of p53 to affect the tumorigenesis [53,54]. According to the results of the betweenness and survival analysis in four disease types, LAMC2, GSN, and HIPK2 may respectively be the potential regulator in cell lines with grade 2 carcinoma, grade 3 carcinoma, and transitional cell carcinoma lymphatic metastasis. On the other hand, we also confirmed the protein expression of these potential regulators (LAMC2, GSN, and HIPK2). Except for HIPK2, we observed the differential protein expression in LAMC2 and GSN. Notably, immunohistochemistry (IHC) data of GSN2 and LAMC2 were coming from the 12 patients in the HPA database, which may not be representative. Therefore, a more stained section of bladder cancer patients was still required for further validation.

In addition, this study provides the potential molecular pathways in different disease types as demonstrated by the results of functional and pathway enrichment analysis. The metabolism of lipids and immune signaling pathways were highly related to human adenocarcinoma lymph node metastasis. In cell lines of grade 2 carcinoma, the dominant process was mitosis via integrin mediating. The role of the integrin could deserve further investigation. On the other hand, the gene expression of grade 3 carcinoma revealed that the PDGF, IGF-1, and TRAIL pathways might be the important molecular mechanism to modulate tumor growth. This requires further investigation because the effects on the tumor in TRAIL pathways were opposite to growth factor signaling pathways. DNA-related process and eNOS activity were associated with the cell lines with transitional cell carcinoma lymphatic metastasis. Most studies used the patient’s expression profile to conduct the WGCNA analysis and identified the module related to the stage [55,56,57,58]. Di et al. [55] identified the THY1, AEBP1, CDH11, COL1A1, COL1A2, COL11A1, MMP2, PXDN, BGN, COL5A1, and COL8A1 as prognostic genes, which had different expression levels in different stages. Chen et al. [56] identified TPST1 and P3H4, which is associated with prognostic according to the differential expression profiles. Besides, Yan et al. [57] also identified the prognostic genes (ASPM, C4orf46, CCNB1, DIAPH3, MLF1, TFR2, NSUN6, and OIP5) with the patient’s transcriptional profiles. On the other hand, Ding et al. [58]. identified the LGALS4 related to progression from WGCNA data and experimental data. Considering the heterogeneity of bladder cancer as the main cause of high recurrence, we choose the expression profile of cell lines to conduct WGCCA analysis for investigating the hub genes of different disease types. On the other hand, we used the clinical data to identified prognostic genes. Based on this pipeline, our results may have higher possibilities to be validated in the in vitro and translated into the in vivo. 

## 4. Materials and Methods 

### 4.1. Dataset Collection

**Figure 11:**
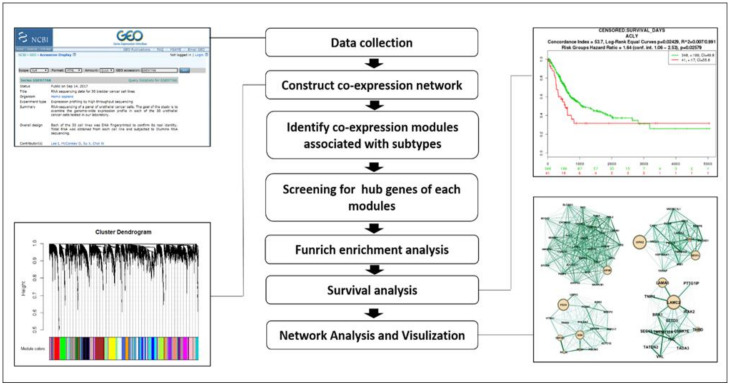
Flowchart of analysis.

**Figure 12:**
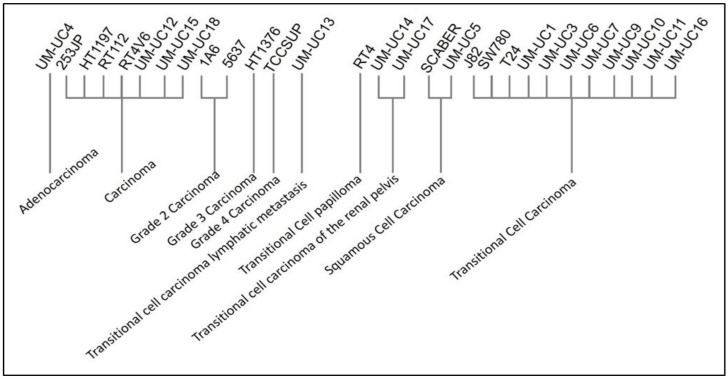
Diseases of bladder cancer cell lines 4.2. Data Preprocessing.

A flowchart of this study is presented in Figure 11. Gene expression profiles of Dataset GSE97768 were downloaded from Gene Expression Omnibus (GEO) database (https://www.ncbi.nlm.nih.gov/geo/query/acc.cgi?acc=GSE97768). This dataset contains the RNA-sequencing profiling of 30 human urothelial cancer cell lines. Except for BV cell lines with unknown origin, we included 29 cell lines, which were clustered according to the disease type (Figure 12). The disease type of human urothelial cancer cell line was retrieved from the American Type Culture Collection (ATCC) and ExPASy Bioformatics Resources Portal [59].

The normalized data was retrieved from the GEO database. All the cell lines were categorized with the disease. The bladder cancer cell line was excluded for unclear information on disease. Ten disease types were identified as following: human adenocarcinoma lymph node metastasis, bladder carcinoma, grade 2 carcinoma, grade 3 carcinoma, grade 4 carcinoma, transitional cell carcinoma lymphatic metastasis, transitional cell papilloma, transitional cell carcinoma of the renal pelvis, squamous cell carcinoma, and transitional cell carcinoma.

### 4.2. Weighted Gene Co-Expression Network Construction

The gene expression data was conducted a scale-free co-expression network with R package: WGCNA [15,60]. WGCNA is an algorithm that is based on high-throughput gene expression profiles, which was best used in gene co-expression network analysis to identification in cancer to reveal the correlation of genes and to search for significantly correlated gene modules. In this study, we used the WGCNA package in R to construct a scale-free co-expression network for the human urothelial cancer cell. The soft threshold power of β = 6 and scale-free R2 = 0.90 was selected to construct a standard scale-free network with pickSoftThreshold function. Then, the network construction and module detection were used by one-step function “blockwiseModules” [61], which constructed the network and detected each module. The parameters of blockwiseModules were implemented with the following: power = 6, maxBlockSize = 6000, minModuleSize = 30, and networkType = “unsigned” in our study.

### 4.3. Finding of Clinical Significant Modules

To find the modules with clinical significance, two approaches were used to identify key modules associated with clinical disease types of bladder cancer. The module eigengenes (MEs) were defined as the first principal component of the gene expression matrix of each corresponding module. The clinical significant module was identified from the correlation between MEs and clinical disease types. Gene significance (GS) is defined as a log10 transformation of the *p*-value (GS = lgP) in the linear regression, which was measured as the correlation between the clinical traits and gene expression. The average GS of all genes in the module is defined as module significance (MS). Modules with a top ranking of absolute MS were selected as candidates related to the clinical disease type. The gray module is defined as null modules, which means some of genes being not clustered to any modules. Considering for comprehensive analysis, we chose to keep the gray module and based on the correlation and analysis of prognosis to check the possible genes.

### 4.4. Candidate Hub Genes Identification

Hub genes in the co-expression network were highly interconnected with nodes in a module, which indicated they have a significant function. The absolute value of the Pearson’s correlation (ModuleMembership > 0.8) was measured as the connectivity of genes. The module membership (MM) was defined as the correlation of module eigengenes (MEs) with gene expression. Thereafter, the hub genes were identified in the module that was highly related to clinical disease subtypes, which were measured by the absolute value of the Pearson’s correlation (GeneSignificance > 0.2). Thus, the hub genes were included based on the following setting: GS > 0.2 and MM > 0.8.

### 4.5. Functional and Pathway Enrichment Analysis

To investigate a comprehensive set of functional annotations of the hub gene, Gene Ontology term enrichment analysis and Kyoto Encyclopedia of Genes and Genomes (KEGG) pathway analysis were performed by using the “FunRich” FunRich was a functional enrichment and interaction network analysis tool, which offered the updating database for performing functional enrichment analysis. GO enrichment analysis and KEGG pathway analysis were performed with the FunRich functional enrichment analysis tool (version 3.1.3). The Top 10 terms with *p* < 0.05 and the number of enrichments more than 2 were selected [62,63].

### 4.6. Network Analysis and Visualization

The analysis of the dynamic nature of gene networks holds remarkable potential in uncovering previously unknown biological phenomena. We performed network analysis by using the R package tidygraph (version: 1.1.2) [64]. The node betweenness centrality (BC) in networks is useful to detect genes with important functional roles [56,65]. BC is defined as the number of shortest paths between every two other nodes in the network that pass through that node. In this study, we chose the brands algorithm to calculate betweenness centrality due to its faster execution. The equation:

(1) $$BC\left(v\right)=\sum_{s\neq v\neq t}^{}\frac{\sigma_{st}\left(v\right)}{\sigma_{st}}.$$

BC is the general formula to determine betweenness centrality for v. v is the set of the gene where σ_st_ represents the number of shortest paths between nodes s and t, while σ_st_ (v) is the number of those paths that pass through gene v. The genes with a high value of BC were indicated as “high traffic genes” and were potentially biologically meaningful. Network visualization and further analysis were performed with Cytoscape (https://cytoscape.org/), which allows visualization, analysis, and interpretation of these networks and helps to better understand the biological association and interactions [65].

### 4.7. The Relationship of Hub Genes and Prognosis

To validate the role of hub genes in the prognosis of bladder cancer, two databases—Survpress and cBioportal—were used to perform the survival analysis [66,67,68]. SurvExpress was a cancer gene expression database providing survival analysis. Data from a total of 390 patients in BLCA-TCGA-Bladder Urothelial Carcinoma (BLCA) was selected. The BLCA patients were divided into low-risk and high-risk groups according to the median value of the prognostic risk score to conduct survival analysis. Those prognostic genes were selected for further analysis with cBioPortal (www.cbioportal.org) and confirmed with The Cancer Genome Atlas (TCGA) database (413 samples). Genes with a *p*-value < 0.05 in survival analyses in Survpress and ciBioportal were defined as the “real” hub genes. Moreover, a gene with the highest betweenness degree was combined with other hub genes to conduct the co-expression survival analysis. 

### 4.8. The Protein Expressions of Prognostic Hub Genes

The Human Protein Atlas (HPA) is an open-access protein expression database, which contains the amount of human transcriptomic and proteomic data in cells, tissues, and organs [69]. The protein expression of the prognostic hub gene between BLCA and normal tissues was validated using immunohistochemistry (IHC) provided by the Human Protein Atlas database (HPA, https://www.proteinatlas.org/).

## 5. Conclusions

We constructed a gene co-expression network and used betweenness centrality to identify and validate the hub genes associated with the prognosis of bladder cancer. The several potential genes and molecular mechanisms in different disease types found in this study require further investigation.

## Supplementary Materials

The following are available online at https://www.mdpi.com/2072-6694/12/7/1809/s1, Table S1: Number of genes in each module, Table S2: Hub gene list of modules that were highly disease-correlated, Figure S1: The top 10 enrichment of gene ontology and pathway for hub genes of cell lines with human adenocarcinoma lymph node metastasis, Figure S2: The top 10 enrichment of gene ontology and pathway for hub genes of cell lines with grade 2 carcinoma, Figure S3: The top 10 enrichment of gene ontology and pathway for hub genes of cell lines with grade 3 carcinoma, Figure S4: The top 10 enrichment of gene ontology and pathway for hub genes of cell lines with transitional cell carcinoma lymphatic metastasis.
