# Supplementary material for: Identification of GSN and LAMC2 as Key Prognostic Genes of Bladder Cancer by Integrated Bioinformatics Analysis
Source: Cancers (Basel). 2020 Jul 6;12(7):1809. doi: 10.3390/cancers12071809 (PMC7408759; doi:10.3390/cancers12071809)
Supplement: Supplementary file 1 [file cancers-12-01809-s001.pdf]

cancers

MDPI

Supplementary Files

Identification of GSN and LAMC2 as Key Prognostic Genes of Bladder Cancer by Integrated Bioinformatics Analysis

Jia-Lian Yang, Charles C.N. Wang, Jia-Hua Cai, Che-Yi Chou, Yu-Chao Lin and Chin-Chuan Hung

Table S1. Number of genes in each module.

Module	The Number of Genes
black	209
blue	330
brown	305
cyan	136
darkgreen	88
darkgrey	83
darkmagenta	46
darkolivegreen	49
darkorange	78
darkred	90
darkturquoise	87
green	215
greenyellow	178
grey	201
grey60	130
lightcyan	134
lightgreen	129
lightyellow	105
magenta	207
midnightblue	136
orange	81
paleturquoise	61
pink	208
purple	182
red	209
royalblue	104
saddlebrown	65
salmon	141
skyblue	67
steelblue	63
tan	152
turquoise	340
violet	59
white	77
yellow	255

Table S2. Hub gene list of moduleswith highlydisease-correlated.

Disease type	Module	Gene
human adenocarcinoma lymph node metastasis	Red	*EPCAM, ACLY, ERO1L, LGALS3, MYO1D, SLC25A1, HMGCS1, CHCHD10, CRKL, EIF5B, MAPK1, GUCD1, KIAA1671, BCR, FKBP4, MVD, PPM1F, ADD3, SMARCB1, SNRPD3, SERBP1, CKB, PI4KA, LZTR1, YDJC, PPIL2, TRMT2A, UBE2L3, CABIN1*
Bladder carcinoma	turquoise	*S100A6, AKR1C3, GPX4, DKK1, CD63, TRAM1, SLC12A7, FPGS, SLC35B2, CDK4, SSR4, UGDH, RAB32, JTB, UBL4A, APOA1BP, ATP6V0B, PGAM5, LAMTOR1, GPRC5B, SYNGR2, SCAMP3, NDUFB11, DCTPP1, AGPAT6, ECI1, CSF1, WDR81, SIVA1, IFRD2, RPS19BP1, STRA13, LRRC61, TMEM141, CHMP2B*
Grade 2 Carcinoma	darkturquoise	*LAMC2, PTTG1IP, LAMA3, TNIP1, TMEM132A, TNC, SETD5, CSNK1E, THBD, SEC13, TADA3, BRK1, IRAK2, VHL, TATDN2*
Grade 3 Carcinoma	lightgreen	*PERP, PCDH1, BCL9L, PDLIM5, PHLDB2, GSN, NDFIP2, BZW2, PSD4, ABCG2, KCTD12, RNF217, USP53, HTRA1, GNAQ, ZNF362*
Grade 4 Carcinoma	magenta	*FLNA, MYH9, ACTB, PTRF, TLN1, TIMP2, ZYX, CALD1, TRAM2, SH3BGRL3, ITGA5, PDLIM7, MAP1B, GDI1, HPCAL1, CUL4B, ACTR1A, TGFB1I1, COL5A2, CPA4, DAPK3, DAB2, SHCBP1, STXBP1, KATNAL1*
transitional cell carcinoma lymphatic metastasis	Royalblue	*CAV1, HIPK2, VGLL3, HSP90AA1, TRRAP, MIB1, TMEM248, SMCHD1, VKORC1L1, RBBP8, SEH1L, SS18, SSBP1, PMPCB, CAPZA2, GUSB*
Transitional Cell papilloma	Salmon	*DTX4, ERBB3, ATP8B1, LLGL2, FOXA1, ARHGAP27, TC2N, PIK3C2B, RAB15, BCAT2, RALGAPA2, C2CD2, TBC1D8, ACSF2, ZNF320, AP1G2, AGR2, FAM59A*
transitional cell carcinoma of the renal pelvis	Grey	*AGPS*
Squamous Cell Carcinoma	Darlorange	*EGFR, GLUL, GCLC, CAT, SLC1A3, SPG11, UFL1, KRT15*
Transitional Cell Carcinoma	Greenyellow	*HNRNPL*

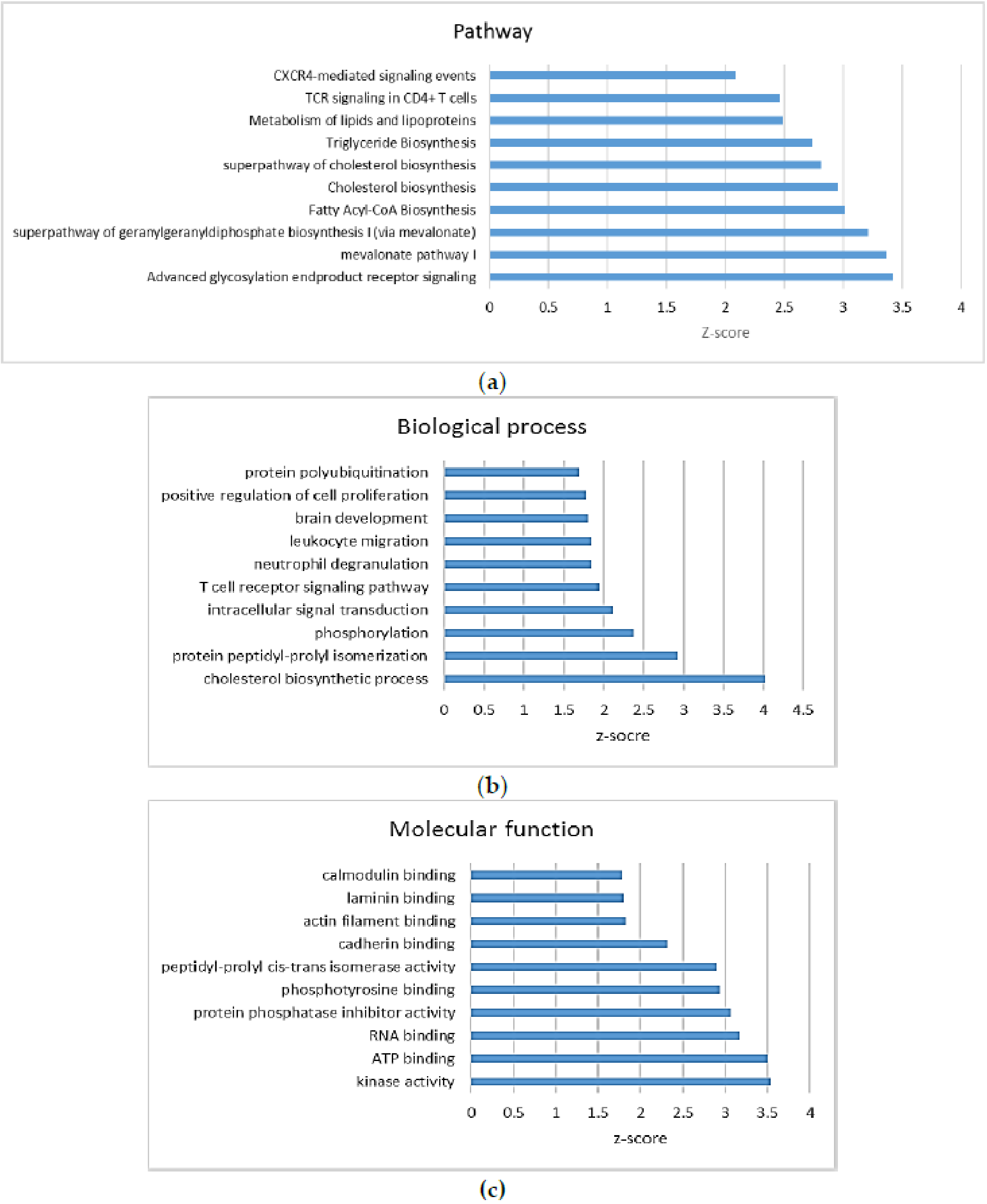

Figure S1. The top 10 enrichment of gene ontology and pathway for hub genes of cell lines with human adenocarcinoma lymph node metastasis. Gene ontology and pathways with z-scores greater than 1.65, which corresponds to a *p*-value 0.1 as statistically significant pathways. Only significant hits with overlap size ≥ 2 (genes that are overlapping in the same pathway) were selected. (**a**) Pathways (**b**) Biological process (**c**) Molecular function.

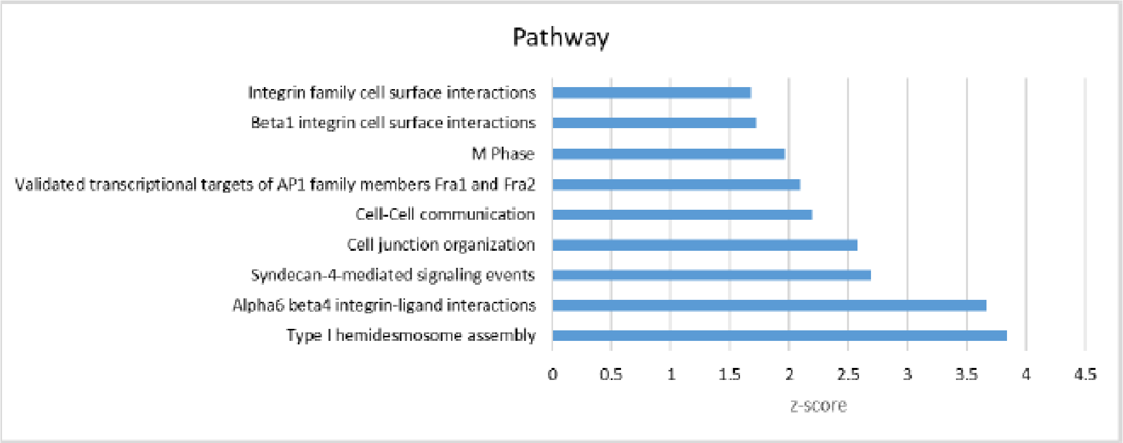

(a)

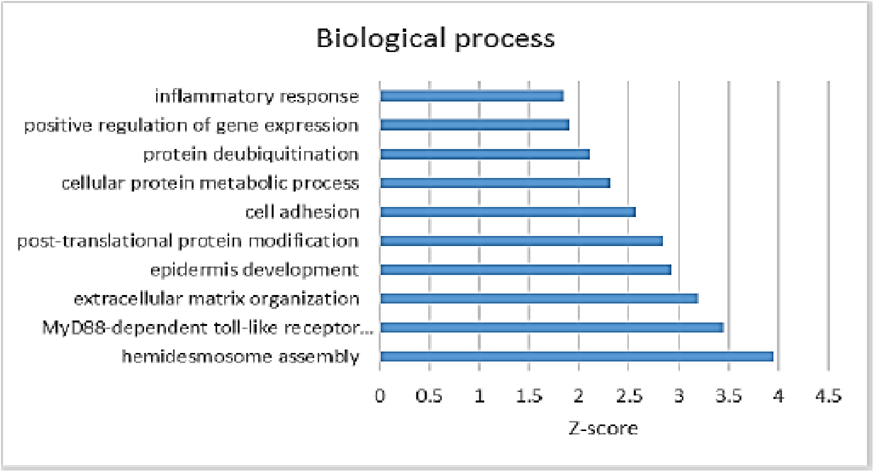

(b)

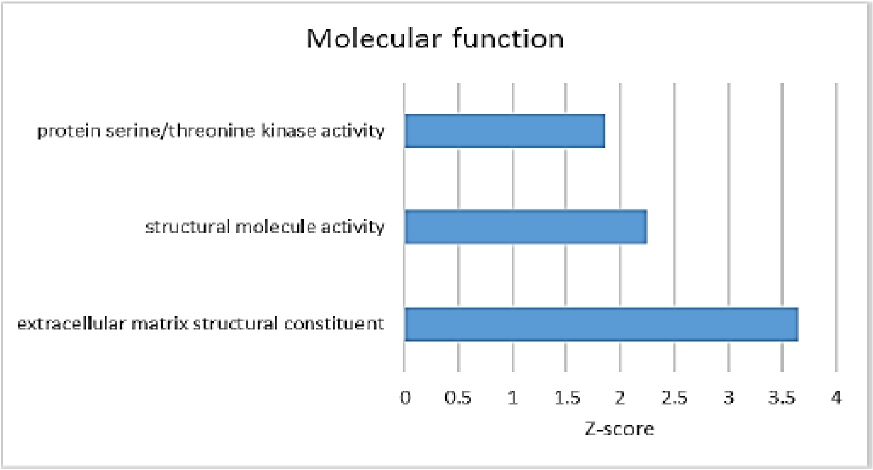

(c)

Figure S2. The top 10 enrichment of gene ontology and pathway for hub genes of cell lines with grade 2 carcinoma. Gene ontology and pathways with *z*-scores greater than 1.65, which corresponds to a *p*-value 0.1 as statistically significant pathways. Only significant hits with overlap size ≥ 2 (genes that are overlapping in the same pathway) were selected. (**a**) Pathways (**b**) Biological process (**c**) Molecular function.

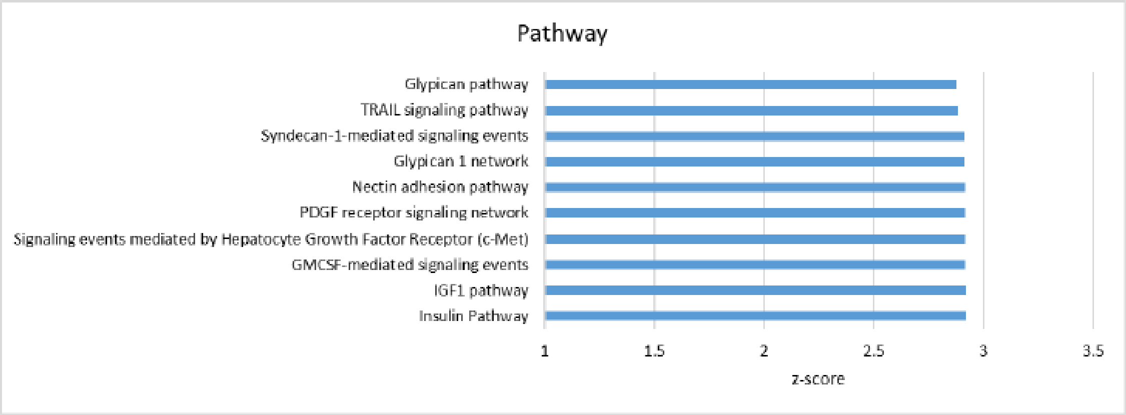

(a)

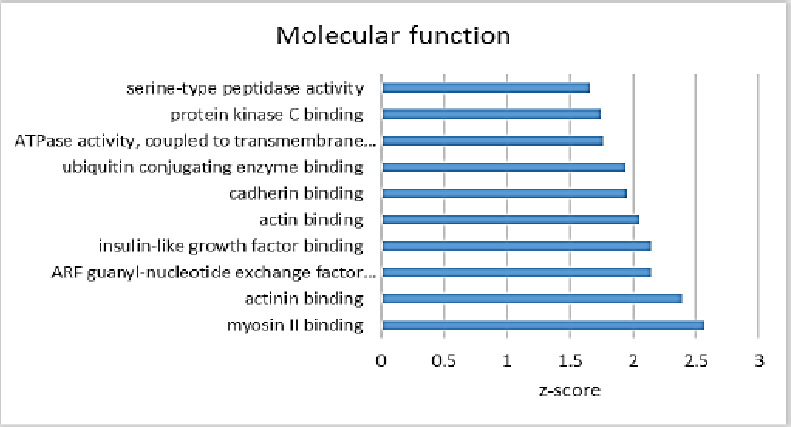

(b)

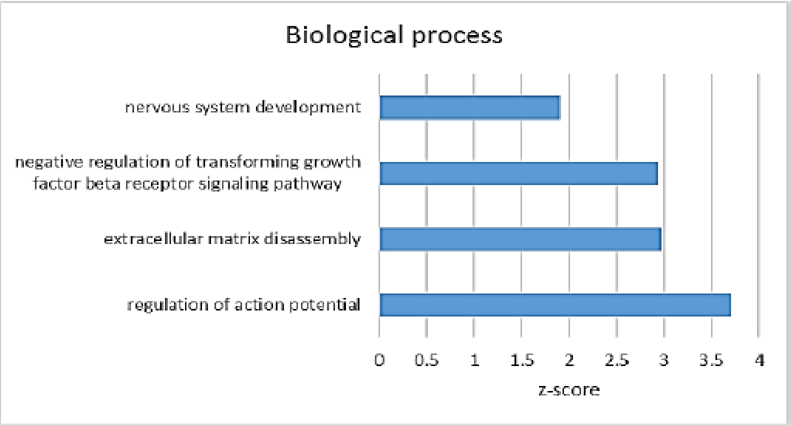

(c)

Figure S3. The top 10 enrichment of gene ontology and pathway for hub genes of cell lines with grade 3 carcinoma. Gene ontology and pathways with *z*-scores greater Table 1. 65, which corresponds to a *p*-value 0.1 as statistically significant pathways. Only significant hits with overlap size ≥ 2 (genes that are overlapping in the same pathway) were selected. (**a**) Pathways (**b**) Biological process (**c**) Molecular function.

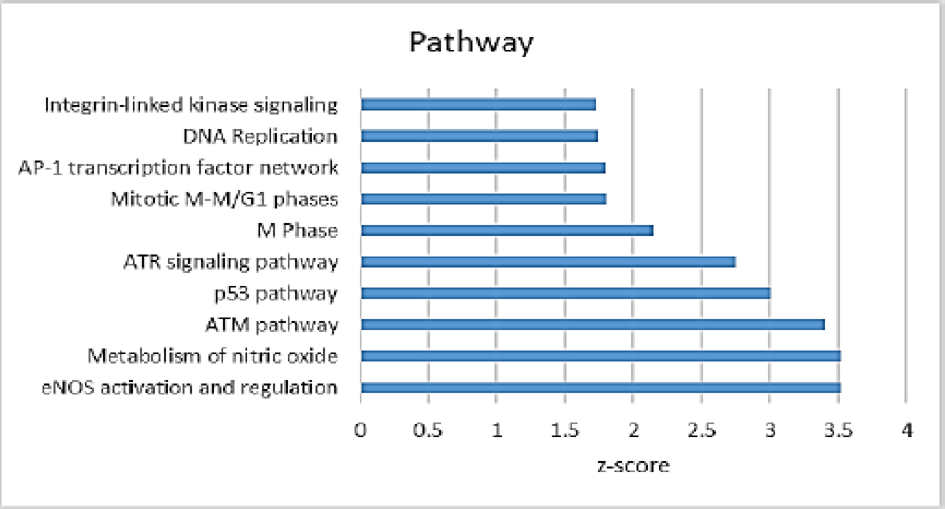

(a)

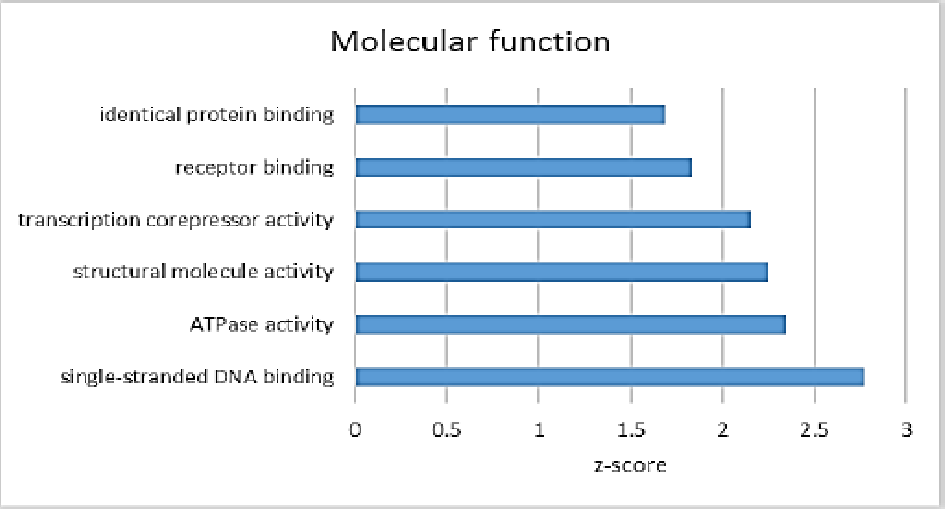

(b)

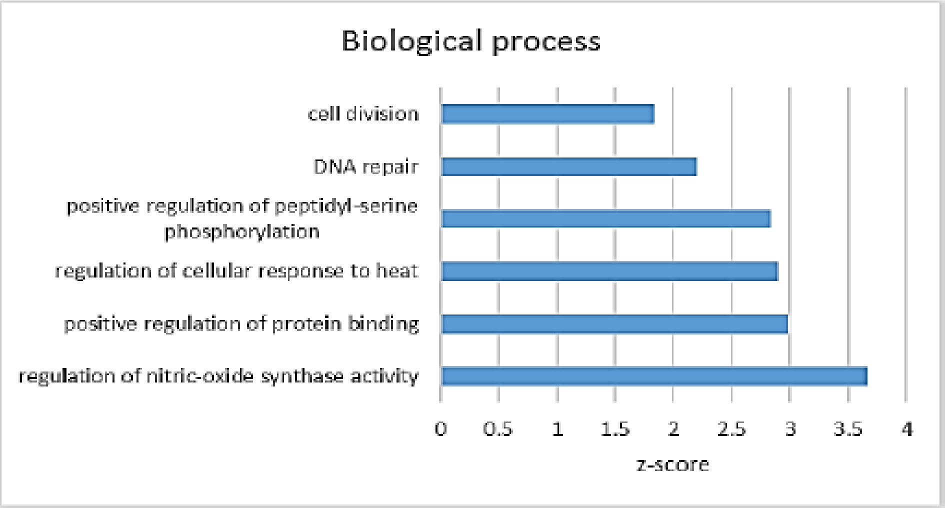

(c)

Figure S4. The top 10 enrichment of gene ontology and pathway for hub genes of cell lines with transitional cell carcinoma lymphatic metastasis. Gene ontology and pathways with *z*-scores greater than 1.65, which corresponds to a **p**-value 0.1 as statistically significant pathways. Only significant hits with overlap size ≥ 2 (genes that are overlapping in the same pathway) was selected. (**a**) Pathways (**b**) Biological process (**c**) Molecular function.

© 2020 by the authors. Licensee MDPI, Basel, Switzerland. This article is an open access article distributed under the terms and conditions of the Creative Commons Attribution (CC BY) license (http://creativecommons.org/licenses/by/4.0/).
